# Real-World Completion of Multitarget Stool DNA Testing Among Texas Medicare Beneficiaries: Associations With Payer Type, Digital Outreach, and Social Vulnerability

**DOI:** 10.36469/001c.162887

**Published:** 2026-06-15

**Authors:** Timothy Ritter, Shrey Gohil, Betthany Thomas, Travelle Ellis, Gustavus Aranda, Sushovan Guha, Mallik Greene

**Affiliations:** 1 GI Alliance Research, Southlake, Texas; 2 Abbott, Madison, Wisconsin; 3 Houston Regional Gastroenterology Institute, Houston, Texas

**Keywords:** colorectal cancer screening, multitarget stool DNA, screening completion, Medicare, real-world evidence, digital outreach, health disparities

## Abstract

**Background:**

Colorectal cancer is a leading cause of cancer-related morbidity and mortality in the United States; however, a substantial proportion of eligible adults do not complete screening.

**Objectives:**

To evaluate real-world completion of multitarget stool DNA (mt-sDNA) testing for colorectal cancer screening among Medicare beneficiaries in Texas and to identify beneficiary-, provider-, and outreach-related factors associated with test completion.

**Methods:**

This retrospective cohort study included Texas beneficiaries aged 65 to 85 years with traditional Medicare or Medicare Advantage coverage who received an mt-sDNA kit between October 2014 and December 2024. Completion was defined as return of a valid test kit within 1 year of shipment. Multivariable logistic regression was used to identify factors associated with completion. Kaplan–Meier and Cox proportional hazards models were used to evaluate time to kit return.

**Results:**

Among 710 515 beneficiaries, 538 377 (75.8%) completed mt-sDNA testing within 1 year. Completion was higher among beneficiaries with traditional Medicare vs Medicare Advantage (81.7% vs 71.7%). In adjusted analyses, gastroenterologist ordering (odds ratio [OR], 2.66; 95% confidence interval [CI], 2.53-2.79), full digital outreach (OR, 1.73; 95% CI, 1.69-1.77), and prior mt-sDNA use (OR, 1.61; 95% CI, 1.59-1.64) were associated with higher odds of completion. Higher neighborhood income and lower social vulnerability were also associated with higher completion rates. Time-to-event analyses were consistent, with faster completion observed among patients receiving digital outreach, with prior mt-sDNA use, and those with gastroenterologist-ordered tests.

**Discussion:**

These findings suggest that both patient context and modifiable care delivery factors influence screening completion and may inform the design of more effective and equitable CRC screening programs.

**Conclusions:**

In this large real-world Medicare cohort, mt-sDNA completion was high overall but varied by payer type, provider specialty, outreach modality, and socioeconomic vulnerability.

## INTRODUCTION

Colorectal cancer (CRC) remains a leading cause of cancer-related morbidity and mortality in the United States.^1^ Screening has been shown to reduce CRC mortality; however, a substantial proportion of eligible adults do not complete recommended screening.^2,3^ Completion of CRC screening tests is an important consideration when evaluating the overall health outcomes of CRC screening, because the effectiveness of screening programs depends on both test performance and patient follow-through. Improving screening completion is therefore an important component of efforts to reduce the burden of CRC at the population level.^4^

CRC screening methods include invasive procedures such as colonoscopy and noninvasive stool-based tests that can be completed at home.^5,6^ Stool-based approaches may reduce logistical barriers associated with in-person procedures and have been associated with higher screening participation in some settings.^4,7–10^ Multitarget stool DNA (mt-sDNA) testing is a noninvasive screening option that has demonstrated strong clinical performance and has been increasingly adopted in routine practice.^11^

Prior US population studies have found that between 50% and 75% of patients who receive mt-sDNA tests complete the test within 1 year.^12–14^ Lower adherence was seen in a Medicaid population (51%),^12^ and a Hispanic population (64%).^14^ Higher adherence was seen in a national sample aged 45-49 years (69%)^15^ and an Asian American population (71%).^13^ Texas has higher CRC incidence and mortality and lower screening rates compared with national averages.^2^ The state also has substantial socioeconomic diversity, including higher poverty rates and lower median income than the United States overall, factors that have been associated with reduced screening uptake.^16–19^ Despite these disparities, limited evidence exists describing real-world completion of stool-based screening tests among Medicare beneficiaries in Texas. Understanding how CRC screening completion varies by payer type, provider specialty, outreach modality, and social risk factors may help inform more efficient and equitable screening program design particularly because improved screening participation has been associated with better CRC outcomes and may reduce downstream healthcare burden.^3^

This study evaluated real-world completion of mt-sDNA testing among Texas Medicare beneficiaries and examined whether payer type, provider specialty, outreach modality, and area-level socioeconomic vulnerability were associated with completion and time to kit return.

## METHODS

### Data Sources

This retrospective study used deidentified laboratory data from Abbott (Madison, Wisc.), the exclusive laboratory provider for mt-sDNA testing in the United States. The dataset included patient demographics, test ordering information, kit shipment and return dates, and insurance payer type.

The study was considered exempt research under 45 CFR § 46.104(d)(4) because it involved secondary analysis of deidentified data in compliance with the Health Insurance Portability and Accountability Act of 1996 (45 CFR § 164.514). The study was conducted in accordance with the Declaration of Helsinki and its later amendments.

### Study Design and Population

This retrospective cohort study included Texas Medicare beneficiaries aged 65 to 85 years who were prescribed an mt-sDNA test between October 1, 2014, and December 31, 2024. Patients were required to have either traditional Medicare or Medicare Advantage coverage at the time of test order.

The index date was defined as the date of mt-sDNA kit shipment. Patients were followed for up to 1 year from the index date to assess completion of the test. Analyses were restricted to patients whose kit shipment date allowed a full 1-year observation window for ascertainment of the primary outcome.

Patients were excluded if they were younger than 65 years or older than 85 years, if the test order did not originate from a point-of-care setting, or if key variables (sex, insurance type, or median household income) were missing.

### Variables and Outcomes

The primary outcome was completion of mt-sDNA testing, defined as return of a valid test kit (positive or negative result) to the laboratory within 1 year of shipment. Time to completion was defined as the number of days from kit shipment to return of a valid kit.

Patient characteristics included age, sex, insurance type (traditional Medicare or Medicare Advantage), urban/rural classification, median annual household income by zip code, and Social Vulnerability Index (SVI) quartile. Urbanicity was classified using Rural-Urban Commuting Area codes from the US Department of Agriculture. Median household income was obtained from the American Community Survey, and SVI measures were obtained from the Centers for Disease Control and Prevention. Area-level income and SVI were used as proxies for socioeconomic context and should be interpreted as neighborhood-level rather than individual-level measures.

Provider specialty (gastroenterologist, primary care physician, nurse practitioner/physician assistant, obstetrician/gynecologist, or other) and patient history of prior mt-sDNA testing were also evaluated. Outreach modality was categorized based on patient communication preferences at the time of order and included full digital (short message service [SMS] and email), partial digital (SMS only or email only), or no digital outreach. Patients receiving digital outreach were sent reminders to encourage kit completion. The unknown outreach category reflects missing or unavailable data on communication preference and does not necessarily indicate absence of outreach.

### Statistical Analysis

Descriptive statistics were used to summarize patient characteristics and mt-sDNA test completion rates. Differences in completion across subgroups were evaluated using chi-square tests. Mann-Whitney *U* and Kruskal-Wallis tests were conducted to assess differences in time to completion.

Multivariable logistic regression was used to estimate adjusted odds ratios (ORs) and 95% confidence intervals (CIs) for factors associated with test completion. Covariates included age, sex, insurance type, urban/rural classification, median annual household income, SVI quartile, provider specialty, outreach modality, and prior mt-sDNA use. Because the SVI incorporates socioeconomic indicators that may overlap conceptually with median household income, additional collinearity diagnostics and sensitivity analyses were performed to assess the potential impact of including both variables in multivariable models. Additional sensitivity analyses were conducted to evaluate the potential impact of the “unknown” outreach category, including assessment of outreach classification patterns across calendar years and multivariable analyses excluding patients with unknown outreach status.

Time to test completion was evaluated using Kaplan-Meier methods, with differences across groups assessed using log-rank tests. Multivariable Cox proportional hazards models were used to estimate adjusted hazard ratios (HRs) and 95% CIs for time to completion.

The proportional hazards assumption was assessed using standard diagnostic methods.

All statistical analyses were performed using SAS version 9.4 (SAS Institute Inc., Cary, N.C.), and a two-sided *P*-value <.05 was considered statistically significant.

## RESULTS

### Study Population

A total of 710 515 Medicare beneficiaries in Texas were included in the study (**Table 1**). Most patients were aged 65 to 75 years, were female, and resided in metropolitan areas. Overall, 75.8% of patients (n=538 377) completed mt-sDNA testing within 1 year of kit shipment (**Table 1**).

**Table 1.** Demographics, mt-sDNA Completion, and Days to Completion Among mt-sDNA Users in Texas Insured by Medicare

**Characteristic**	**n (%)**	**mt-sDNA Test Completion, n (%)**	**Days to Completion, Median (IQR)**
Overall	710 515 (100)	538 377 (75.8)	15 (10-23)
Age, years		*P* < .001	*P* < .001
65-75	581 935 (81.9)	437 354 (75.2)	15 (10-24)
76-85	128 580 (18.1)	101 023 (78.6)	14 (9-21)
Sex		*P* < .001	*P* < .001
Female	426 525 (60.0)	323 946 (76.0)	15 (10-24)
Male	283 990 (40.0)	214 431 (75.5)	14 (9-22)
Health insurance payer		*P* < .001	*P* < .001
Medicare Advantage	422 070 (59.4)	302 684 (71.7)	15 (10-23)
Medicare	288 445 (40.6)	235 693 (81.7)	14 (10-22)
Urban/rural classification		*P* < .001	*P* < .001
Metropolitan	596 080 (83.9)	449 578 (75.4)	15 (10-23)
Micropolitan	69 496 (9.8)	54 252 (78.1)	14 (10-22)
Small town	31 658 (4.5)	24 334 (76.9)	15 (10-23)
Rural	13 269 (1.9)	10 203 (76.9)	15 (11-24)
Unknown	12 (<0.01)	10 (83.3)	15 (13-16)
Median annual household income by zip code, $		*P* < .001	*P* < .001
<50 000	84 150 (11.8)	56 208 (66.8)	15 (10-24)
50 000-75 000	280 286 (39.5)	208 448 (74.4)	15 (10-23)
75 000-100 000	181 789 (25.6)	141 652 (77.9)	14 (10-23)
100 000-125 000	108 484 (15.3)	86 476 (79.7)	14 (10-23)
≥125 000	55 806 (7.9)	45 593 (81.7)	14 (10-23)
Social Vulnerability Index quartile		*P* < .001	*P* < .001
Least vulnerable	140 516 (19.8)	114 795 (81.7)	14 (10-22)
Less vulnerable	136 757 (19.3)	107 690 (78.8)	14 (10-23)
More vulnerable	159 066 (22.4)	120 041 (75.5)	15 (10-23)
Most vulnerable	197 721 (27.8)	135 764 (68.7)	15 (10-23)
Unknown	76 455 (10.8)	60 087 (78.6)	15 (11-24)
Provider specialty^a^		*P* < .001	*P* < .001
GI	17 743 (2.5)	15 692 (88.4)	13 (9-20)
NP/PA	135 317 (19.1)	96 710 (71.5)	15 (10-23)
OB/GYN	7811 (1.1)	6287 (80.5)	15 (11-23)
PCP	515 477 (72.8)	394 558 (76.5)	15 (10-23)
Other	31 876 (4.5)	23 326 (73.2)	15 (10-23)
Type of outreach		*P* < .001	*P* < .960
Full digital	131 771 (18.6)	106 272 (80.7)	14 (10-22)
No digital	83 477 (11.8)	56 266 (67.4)	14 (10-25)
Email only	20 895 (2.9)	16 090 (77.0)	14 (10-23)
SMS only	302 393 (42.6)	218 166 (72.2)	15 (10-23)
Unknown	171 979 (24.2)	141 583 (82.3)	14 (9-23)
Patient test kit return history		*P* < .001	*P* < .960
≥1 prior returns	151 253 (21.3)	124 065 (82.0)	15 (10-23)
New patient	559 262 (78.7)	414 312 (74.1)	15 (10-23)

Abbreviations: GI, gastroenterologist; IQR, interquartile range; mt-sDNA, multitarget stool DNA; NP/PA, nurse practitioner/physician assistant; OB/GYN, obstetrician/gynecologist; PCP, primary care physician; SMS, short message service.

^a^Provider specialty information was unavailable for 2291 patients. Percentages reflect available data.

### Completion Rates by Demographic Category

Completion differed by insurance type, with higher completion observed among beneficiaries with traditional Medicare compared with Medicare Advantage (81.7% vs 71.7%) (**Table 1**). Completion varied substantially by outreach modality. Patients receiving full digital outreach had higher completion compared with those receiving no digital outreach (80.7% vs 67.4%). Partial digital outreach demonstrated intermediate completion rates (**Table 1**). Additional analyses demonstrated that the “unknown” outreach category was concentrated primarily in earlier calendar years (2014-2020), whereas structured digital outreach categories became substantially more prevalent beginning in 2020-2021. Adherence rates within the unknown category were highest during the earliest study years and declined in later years, suggesting that the category likely reflected historical documentation practices rather than a distinct outreach strategy. Sensitivity analyses excluding patients with unknown outreach status demonstrated minimal changes in the magnitude or direction of outreach-related effect estimates, supporting the robustness of the primary findings regarding digital outreach and mt-sDNA completion.

Provider specialty was strongly associated with completion. Patients whose tests were ordered by gastroenterologists had the highest completion (88.4%), followed by primary care physicians (76.5%), while lower completion was observed for orders placed by nurse practitioners/physician assistants (71.5%).

Completion increased with higher median annual household income and lower social vulnerability. Patients residing in zip codes with median annual household incomes ≥$125 000 had higher completion compared with those in areas with income <$50 000 (81.7% vs 66.8%). Similarly, patients in the least vulnerable SVI quartile had higher completion than those in the most vulnerable quartile (81.7% vs 68.7%).

Patients with prior mt-sDNA test completion had higher completion compared with new users (82.0% vs 74.1%).

### Multivariable Analysis of Predictors of Completion

**Figure 1.**
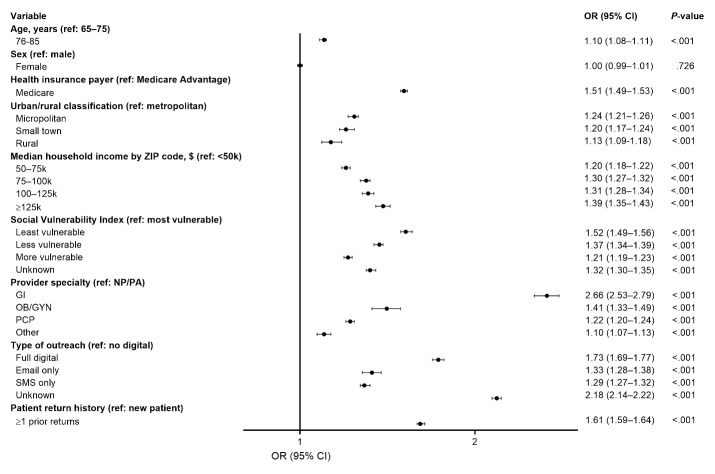
Logistic Regression Analysis of Factors Associated with mt-sDNA Test Completion Among Texas Medicare Beneficiaries Abbreviations: CI, confidence interval; GI, gastroenterologist; mt-sDNA, multitarget stool DNA; NP/PA, nurse practitioner/physician assistant; OB/GYN, obstetrician/gynecologist; OR, odds ratio; PCP, primary care physician; SMS, short message service. The *x*-axis is depicted on a log base 2 scale.

In adjusted analyses, several factors were independently associated with higher odds of completion (**Figure 1**). Gastroenterologist ordering was associated with the greatest increase in odds of completion compared with nurse practitioner/physician assistant ordering (OR, 2.66; 95% CI, 2.53-2.79). Traditional Medicare insurance was associated with higher odds of completion compared with Medicare Advantage (OR, 1.51; 95% CI, 1.49-1.53). Full digital outreach was associated with higher odds of completion compared with no digital outreach (OR, 1.73; 95% CI, 1.69-1.77), and prior mt-sDNA test use was also associated with increased odds (OR, 1.61; 95% CI, 1.59-1.64). Higher neighborhood income and lower social vulnerability were also associated with increased odds of completion (**Figure 1**).

Additional collinearity diagnostics demonstrated a moderate association between median household income and SVI quartile (Cramer’s V = 0.32; *P* < .0001). Sensitivity analyses excluding either median household income or SVI from the multivariable models demonstrated minimal changes in effect estimates and overall model interpretation, suggesting that inclusion of both variables did not introduce substantial multicollinearity or meaningfully distort regression coefficients.

### Time to Completion

**Figure 2.**
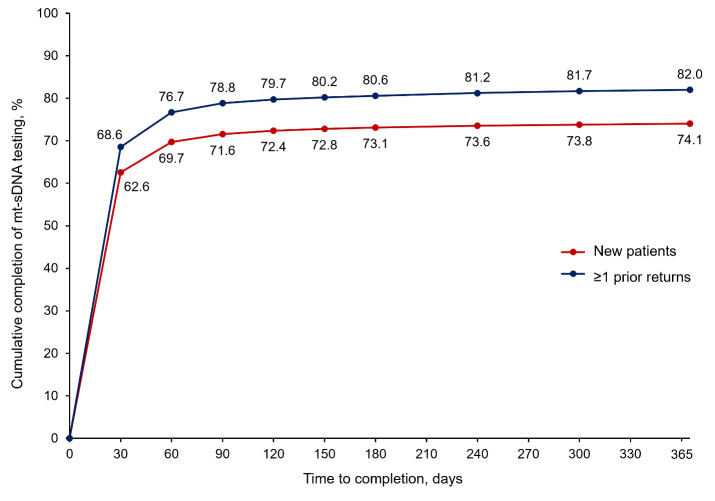
Time to Test Completion Among Texas Medicare Beneficiaries Prescribed an mt-sDNA Test Abbreviation: mt-sDNA, multitarget stool DNA.

Among patients who completed testing, the median time to kit return was 15 days (interquartile range, 10-23 days) (**Table 1**). Approximately 71.6% of new users and 78.8% of prior users completed testing within 90 days of shipment (**Figure 2**).

**Figure 3.**
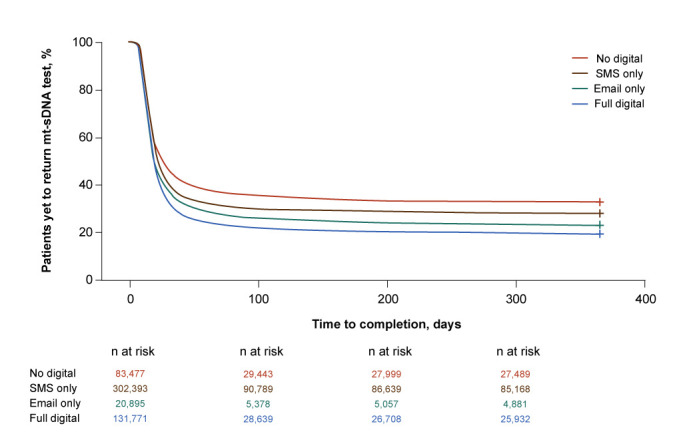
Time to mt-sDNA Test Completion by Type of Outreach Among Texas Medicare Beneficiaries Abbreviations: mt-sDNA, multitarget stool DNA; SMS, short message service. Kaplan-Meier analysis. Log-rank P value calculated across all outreach categories, including “unknown”; the “unknown” category is not shown for visual clarity. Log-rank P value <.001.

**Figure 4.**
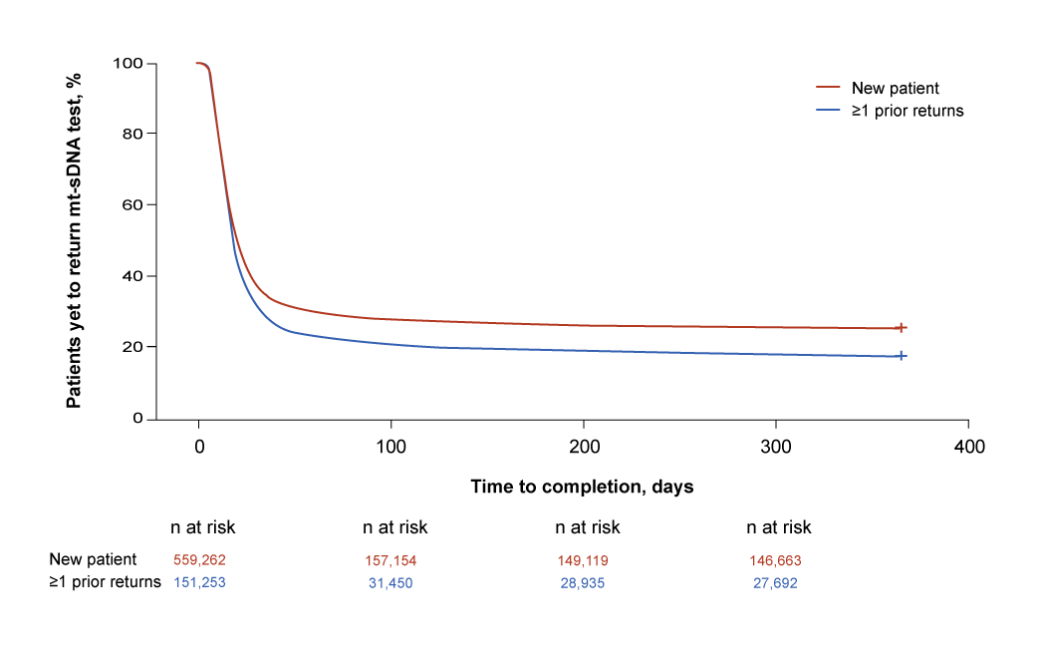
Time to mt-sDNA Test Completion by Patient Return History Among Texas Medicare Beneficiaries Abbreviation: mt-sDNA, multitarget stool DNA. Kaplan-Meier analysis. Log-rank *P* value <.001.

Kaplan-Meier analyses demonstrated differences in time to completion by outreach modality and prior mt-sDNA use, with faster test returns observed among patients receiving digital outreach and those with prior testing history (log-rank *P* < .001 for both analyses) (**Figure 3** and **Figure 4**).

**Figure 5.**
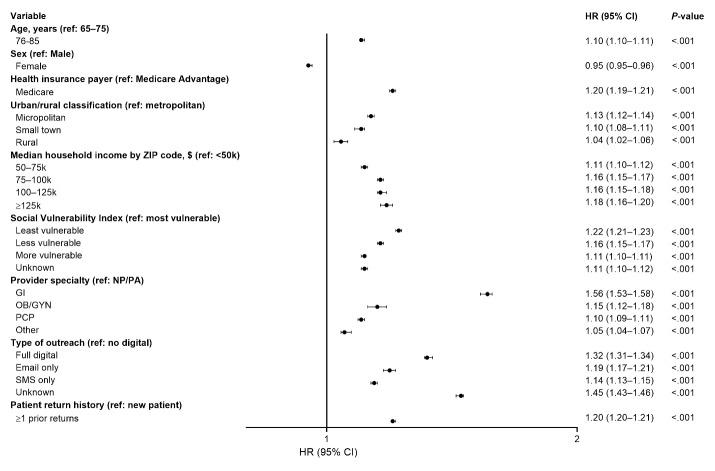
Factors Associated with Time to mt-sDNA Test Completion Among Texas Medicare Beneficiaries Abbreviations: HR, adjusted hazard ratio; CI, confidence interval; GI, gastroenterologist; NP/PA, nurse practitioner/physician assistant; OB/GYN, obstetrician/gynecologist; PCP, primary care physician; SMS, short message service.

In multivariable Cox models, gastroenterologist ordering (HR, 1.56; 95% CI, 1.53-1.58), full digital outreach (HR, 1.32; 95% CI, 1.31-1.34), and prior mt-sDNA use (HR, 1.20; 95% CI, 1.20-1.21) were associated with shorter time to completion (**Figure 5**). Traditional Medicare insurance was also associated with faster completion compared with Medicare Advantage (HR, 1.20; 95% CI, 1.19-1.21) (**Figure 5**).

## DISCUSSION

In this large retrospective cohort of more than 700 000 Texas Medicare beneficiaries, 75.8% of participants completed mt-sDNA testing within 1 year of kit shipment. Completion was consistently higher among beneficiaries with traditional Medicare, whose tests were ordered by gastroenterologists, those receiving digital outreach, and those with prior mt-sDNA experience. In addition, area-level socioeconomic factors, including household income and social vulnerability, were strongly associated with both completion and time to kit return.

From a health outcomes and healthcare delivery perspective, these findings suggest that completion of stool-based screening in Medicare populations is influenced not only by beneficiary characteristics but also by modifiable system-level factors. In particular, the strong association between digital outreach and higher completion highlights the potential role of communication strategies in improving screening participation. Similarly, differences observed by provider specialty suggest that care pathways and provider engagement may influence beneficiary follow-through with screening recommendations. However, the finding that gastroenterologist-ordered tests were more likely to be completed could represent a form of selection bias; patients being seen by gastroenterologists may have greater awareness of gastrointestinal disorders and may be more motivated to complete screening. These findings may be particularly relevant for health systems and payers seeking to optimize CRC screening programs through targeted outreach and improved care coordination.

Although this study did not directly evaluate economic outcomes, improved CRC screening completion may have important downstream clinical and healthcare utilization implications. Prior studies have demonstrated that CRC screening is associated with reduced CRC mortality,^3^ and modeling analyses have suggested that stool-based screening strategies, including mt-sDNA testing (and associated outreach strategies),^20^ can be cost-effective when screening adherence is maintained.^21^ These findings underscore the potential population health relevance of interventions that improve screening participation and timely test completion.

Differences by insurance type are also notable. Beneficiaries enrolled in traditional Medicare had higher completion and faster time to kit return compared with those enrolled in Medicare Advantage plans. Although this study was not designed to evaluate causal mechanisms, these findings may reflect differences in care access, referral patterns, or administrative processes between plan types. Further research is needed to better understand how payer-related factors influence screening completion in real-world settings.

Socioeconomic gradients observed in this study are consistent with prior literature demonstrating lower screening uptake among populations with greater social vulnerability and lower income.^18,19^ These findings highlight ongoing disparities in preventive care and suggest that targeted outreach or tailored interventions may be needed to improve screening completion in more vulnerable populations.

The association between prior mt-sDNA use and higher completion is also notable and may reflect familiarity with the testing process or prior engagement with screening. In addition, the relatively short median time to kit return and high proportion of returns within 90 days suggest that most beneficiaries who complete testing do so soon after kit receipt, emphasizing the importance of early engagement and timely outreach.^22,23^

Completion rates observed in this cohort compare favorably with previously reported CRC screening uptake estimates in the literature^8,22,24–29^; however, cross-study comparisons should be interpreted cautiously due to differences in populations and study designs.

### Limitations

This study has several limitations. First, as a retrospective observational analysis, factors such as beneficiary preferences, health literacy, mobility, functional status, comorbidity burden, and prior screening behavior that were not measured could not be controlled for. Second, income and social vulnerability were measured at the zip code level and may not reflect the socioeconomic status of an individual beneficiary. Third, the study did not compare mt-sDNA completion with completion of other CRC screening modalities. Fourth, the analysis was limited to Medicare beneficiaries in Texas and may not be generalizable to other populations or geographic regions. Fifth, the study did not evaluate downstream clinical outcomes, healthcare utilization, or economic endpoints associated with screening completion. Therefore, the potential cost-effectiveness or long-term clinical impact associated with higher mt-sDNA completion rates could not be assessed in this analysis. Finally, almost 25% of study participants had unknown digital outreach status, which was concentrated primarily in earlier calendar years and likely reflected evolving outreach documentation practices over time. Although sensitivity analyses excluding this group demonstrated minimal changes in outreach-related effect estimates, non-random missingness in outreach classification may still have influenced findings related to communication modality.

## CONCLUSIONS

In this large real-world cohort of Texas Medicare beneficiaries, completion of mt-sDNA testing was high overall but varied meaningfully by payer type, provider specialty, outreach modality, and socioeconomic vulnerability. These findings suggest that both beneficiary-level factors and modifiable care delivery characteristics influence CRC screening completion. Targeted outreach strategies, particularly digital communication approaches, and attention to socioeconomic disparities may help improve screening uptake and support more effective and equitable population-level screening programs.

### Declarations

S.G., B.T., T.E., G.A., and M.G. are employees of Abbott and own company stocks. T.R. and S.G. report nothing to disclose.

## Data Availability

The data that support the findings of this study are available from Abbott and may be made available by the corresponding author upon reasonable request and with appropriate permissions.
